# The Fate of the Outflow Tract Septal Complex in Relation to the Classification of Ventricular Septal Defects

**DOI:** 10.3390/jcdd6010009

**Published:** 2019-02-21

**Authors:** Robert H. Anderson, Justin T. Tretter, Diane E. Spicer, Shumpei Mori

**Affiliations:** 1 Institute of Genetic Medicine, Newcastle University, Newcastle-upon-Tyne NE1 3BZ, UK; 2 Heart Institute, Cincinnati Children’s Hospital Medical Center, Cincinnati, OH 45229, USA; justin.tretter@cchmc.org; 3 Division of Pediatric Cardiology, University of Florida, Gainesville, FL 32611, USA; spicerpath@hotmail.com; 4 Division of Cardiovascular Medicine, Department of Internal Medicine, Kobe University Graduate School of Medicine, Kobe 650-0017, Japan; shumpei_8@hotmail.com

**Keywords:** outflow tract, outflow cushions, aortopulmonary septum, ventricular septum, ventricular septal defects

## Abstract

It is now established that the entity often described as an “aortopulmonary septal complex” is better considered as an “outflow tract septal complex”. This change is crucial for appropriate understanding of not only malformations of the outflow tract, but also ventricular septal defects. Thus, the embryonic outflow tract, as it develops, is separated into its two components by fusion of a protrusion from the dorsal wall of the aortic sac with the distal end of the outflow cushions. The key point with regard to morphogenesis is that, with ongoing development, these structures lose their septal integrity, although they can still be identified as septal structures when the ventricular septum itself is deficient. In the normal postnatal heart, however, the aortic and pulmonary components have their own walls throughout the length of the outflow tracts. All of this is of clinical significance, since some current concepts of categorisation of the ventricular septal defects are based on the existence in the normal heart of a “conal septum”, along with a “septum of the atrioventricular canal”. In this review, we show how analysis of postnatal hearts reveals the definitive ventricular septum to possess only muscular and fibrous components in the absence of either discrete outflow or inlet components. We also show that this information regarding development, in turn, is of major significance in determining whether categorisation of ventricular septal defects is best approached, in the first instance, on the basis of the borders of the defects or the fashion in which they open to the right ventricle.

## 1. Introduction

In a recent review published in the Journal, Poelmann and Gittenberger de Groot discussed in depth the influence of hemodynamics on cardiac development [1]. When describing their experiments, they explained how ligating the right vitelline vein in the developing chick embryo induced the formation of congenital cardiac malformations, including ventricular septal defects. Their findings revealed that the deficient ventricular septation was the result of failure of fusion and muscularisation of the proximal outflow cushions. In this context, they then showed that although the proximal components had failed to fuse, the aortic and pulmonary roots, along with the intrapericardial arterial trunks, had properly separated one from the other. In their discussion, they alluded to the fact that the structures separating the components of the outflow tract at one stage had been described as the “aortopulmonary septal complex” [2]. They then indicated that the more appropriate term was “outflow tract septal complex”. In their subsequent discussion, they provide an exemplary account of the patterns of gene expression involved in mechanosensing and relate these changes to their experimental findings. They do not, however, discuss the potential clinical significance of their appropriate change in terminology from “aortopulmonary septal complex” to “outflow tract septal complex”. Although seemingly a minor change, its implications are crucial for the discussions which are ongoing regarding the most appropriate categorisation of ventricular septal defects when observed in the clinical situation [3]. In our review, therefore, we point to the significance of appropriate understanding of the separation of the components of the developing outflow tract. We emphasise that in the postnatal setting, there are no septal structures interposing between the cavities of the right and left ventricular outflow tracts. These findings regarding the structure of the normal heart are based on the availability of computed tomographic datasets from otherwise normal individuals undergoing assessment at Kobe University for suspected coronary arterial disease. We have recently used these datasets to provide an extensive account of the overall anatomy of the normal heart, including again details of the structural arrangement of the outflow tracts [4]. We have also examined in detail the normal heart specimens held in the cardiac archive of Cincinnati Children’s Hospital. We interrogated one heart using high-resolution magnetic resonance imaging prior to its subsequent dissection. As we show, this permitted us to demonstrate the precise relationships of the components of the ventricular mass. Our findings related to cardiac development are based on our analysis of a large number of datasets obtained from developing mice and prepared using episcopic microscopy [5]. For the key stages of development, involving the period from embryonic days 11.5 through 14.5, we have access to at least 40 datasets for each day of murine development. These datasets were all prepared by Dr Timothy Mohun, and we are grateful to him for making them available to us for these studies. The details regarding the datasets are provided on the website of “Deciphering the Mechanisms of Developmental Disorders” (DMDD). As explained by Dr Mohun on his own website, “all DMDD data is available to view and study online via our database, which is an ever-expanding resource for developmental biologists and clinicians”.

## 2. Significance of Developmental Concepts to Categorisation of Ventricular Septal Defects

**Figure 1:**
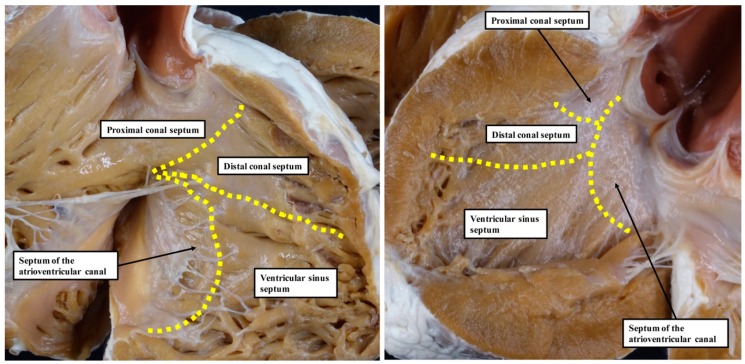
These images, of a normal heart held in the pathological archive of Cincinnati Children’s Hospital, show the suggested division of the definitive ventricular septum based on the presumptive development as described by Van Praagh and his colleagues [5]. The **left**-hand panel shows the view of the opened right ventricle with the lines superimposed as described by Van Praagh and his associates. The **right**-hand panel shows the view from the left ventricle.

One of the systems currently used for the categorisation of ventricular septal defects [6] is based on the notion that, during development, the ventricular septum is derived from four different sources. These components were described as the septum of the atrioventricular canal, the ventricular sinus septum, and the proximal and distal components of the outlet septum (Figure 1).

**Figure 2:**
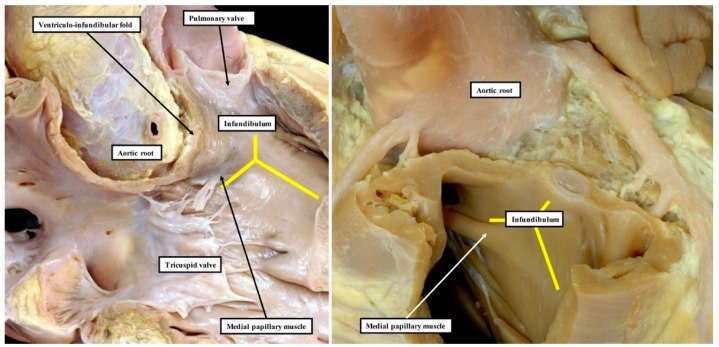
These images show the findings subsequent to the dissection of normal hearts. The image in the **left**-hand panel shows the area of extracavitary adipose tissue that interposes between the posterior wall of the infundibulum, also known as the ventriculo-infundibular fold, and the aortic root. The yellow “Y” shows the septal band, or septomarginal trabeculation, with the medial papillary muscle, or papillary muscle of the conus, arising from its postero-caudal limb. The **right**-hand panel shows another heart in which the myocardial sleeve supporting the leaflets of the pulmonary valve has been sectioned, permitting the pulmonary root to be lifted away from the base of the ventricular mass (see also Figure 4C). The yellow “Y” again shows the location of the septal band. The dissections reveal the lack in the normal heart of a “muscular outlet septum”.

Our own studies, based on extensive examination of normal hearts in the autopsy room (Figure 2), do not support these interpretations.

**Figure 3:**
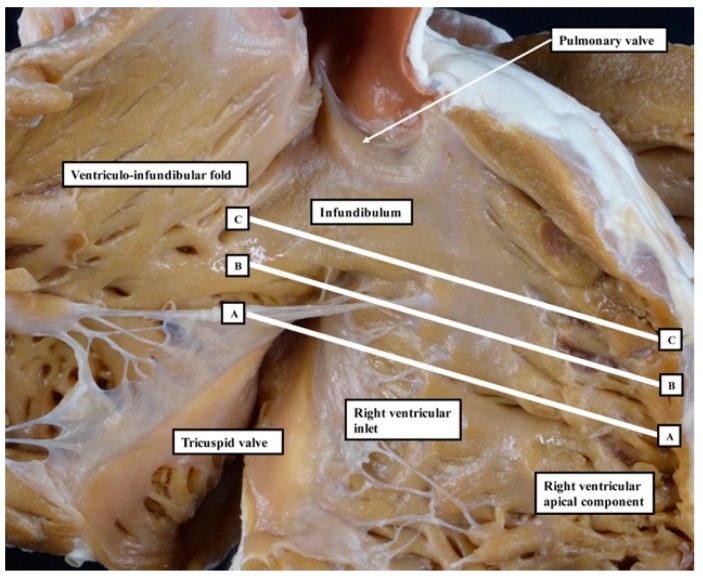
This image shows the right ventricle of the same heart as was used to prepare Figure 1. The heart was scanned using a magnetic resonance scanner of 7 Tesla strength prior to its dissection. We show images in the planes A–A, B–B, and C–C of Figure 3 as obtained subsequent to scanning.

**Figure 4:**
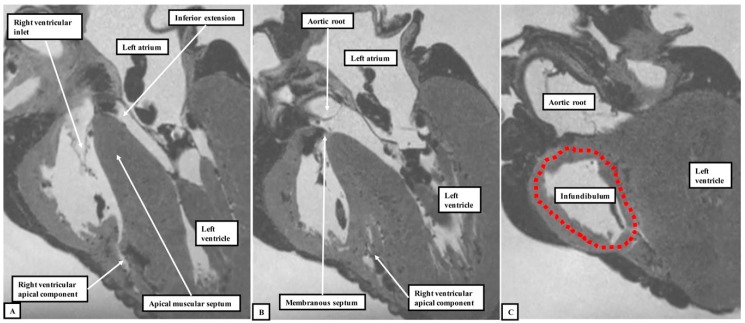
Panels **A** through **C** show the images obtained by scanning the heart photographed in Figure 1 and Figure 3 using magnetic resonance imaging prior to subsequent dissection. They show that, by virtue of the inferior extension of the subaortic outflow tract, the apical muscular ventricular septum separates the inlet of the right ventricle from the outlet of the left ventricle. They also reveal that the subpulmonary infundibulum is a myocardial sleeve, as shown by the dotted red oval in panel **C**.

We have now validated our examinations made on the basis of dissection by using high-resolution magnetic resonance imaging of an intact autopsy specimen prior to its subsequent dissection. Thus, in Figure 3, we show the view of the right ventricle of the same heart we used to produce Figure 1. In Figure 4, we show sectional images through the heart obtained from the three-dimensional dataset of the heart produced by interrogation using a 7 Tesla magnetic resonance scanner prior to dissection. 

**Figure 5:**
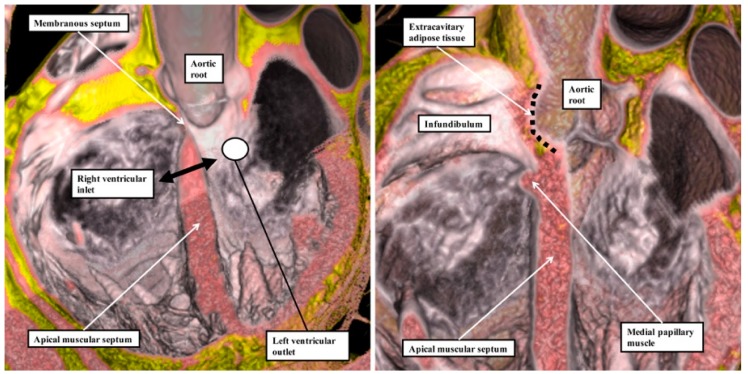
These images were prepared by virtual dissection of a cardiac computed tomographic dataset. The **left**-hand panel shows that the basal and inferior part of the ventricular septum interposes between the inlet of the right ventricle and the subaortic outlet of the left ventricle. The **right**-hand panel shows that the infundibulum of the right ventricle is a free-standing myocardial sleeve. By virtue of the presence of this free-standing sleeve, there is no “outlet septum” in the normal heart.

We then provided further information regarding the inter-relationships of the ventricular components by interrogating computed tomographic datasets obtained during life from individuals undergoing assessment for suspected coronary arterial disease (Figure 5).

The images confirm that there is no “outlet septum” to be found in the normal heart. Instead, it is a free-standing infundibular sleeve which lifts the leaflets of the pulmonary valve away from the base of the left ventricle (Figure 4C). It is the presence of this infundibular sleeve that makes possible the surgical procedure known as the Ross operation [7]. The images also show that there is no “septum of the atrioventricular canal” interposed between the inlets of the right and left ventricles. The inferior component of the muscular ventricular septum, by virtue of the wedged location of the aortic root, interposes between the inlet of the right ventricle and the outlet component of the left ventricle. 

**Figure 6:**
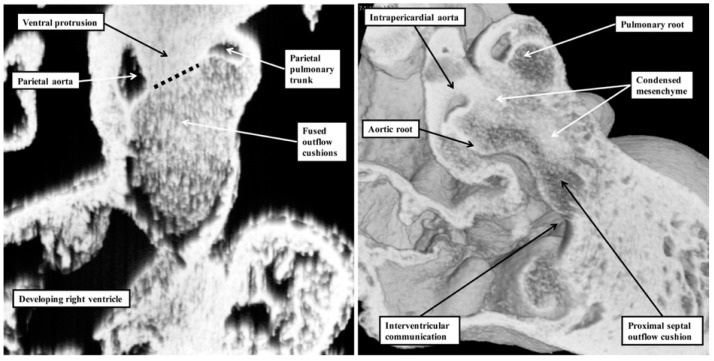
These images show the changes occurring in the separation of the outflow tract into its pulmonary and aortic components in the developing mouse. The **left**-hand panel shows the arrangement at embryonic day 11.5. The ventral protrusion from the dorsal wall of the aortic sac is separating the distal part of the outflow tract into its aortic and pulmonary components. At this stage of development, it is a true aortopulmonary septum. It is the major outflow cushions that are separating the intermediate and proximal components of the outflow tract. The **right**-hand panel shows the arrangement at embryonic day 12.5. By this stage, the distal outflow tract has been divided into the intrapericardial arterial trunks. By this stage, each arterial trunk has its own walls. Only the intrapericardial aorta is seen in this image. The major cushions have fused to separate the intermediate part of the outflow tract into the arterial roots. The proximal parts of the major cushions, however, have still to fuse. They will eventually separate the ventricular outflow tracts. Note the presence of the column of condensed mesenchyme extending into the proximal part of the septal outflow cushion. Said to be part of the “aortopulmonary septal complex”, the columns of condensed mesenchyme play no part in separating the intrapericardial components of the arterial trunks. Instead, they are key to separating the developing arterial roots and ventricular outflow tracts. As was indicated by Poelmann and Gittenberger-de Groot [1], it is more appropriate to describe the combined structures as the “outflow tract septal complex”.

It is the lack of any “outlet septum” in the normal heart that is directly relevant to the appropriate change in terminology from “aortopulmonary septal complex” to “outflow tract septal complex” as proposed by Poelmann and Gittenberger-de Groot [1]. Our investigation of datasets prepared from developing mice reveals that the real aortopulmonary septum separates only the intrapericardial arterial trunks (Figure 6).

With ongoing development, it is the outflow cushions, rather than the aortopulmonary septum, which fuse to separate the developing aortic and pulmonary roots and the ventricular outflow tracts. Although initially having a septal function, these structures cease to interpose between the outflow channels subsequent to closure of the embryonic interventricular foramen. This is because, once separated, the aortic and pulmonary roots develop their own discrete walls, with no septal structures interposing between them. With the passage of time, the proximal parts of the outflow cushions also fuse and then muscularise. Subsequent to closure of the interventricular communication, the muscularised entities lose their septal function, becoming transformed into the free-standing muscular infundibular sleeve. Thus, by the time of birth, there are no septal entities interposing between the entire lengths of the right and left ventricular outflow channels. This means that although the structures are appropriately described as the “outflow tract septal complex” during the period of intrauterine development [1], they can no longer accurately be described in this fashion in postnatal life. As we will show, nonetheless, the tissues derived by muscularisation of the proximal outflow can still be recognised as a myocardial outlet septum in the setting of deficient ventricular septation.

**Figure 7:**
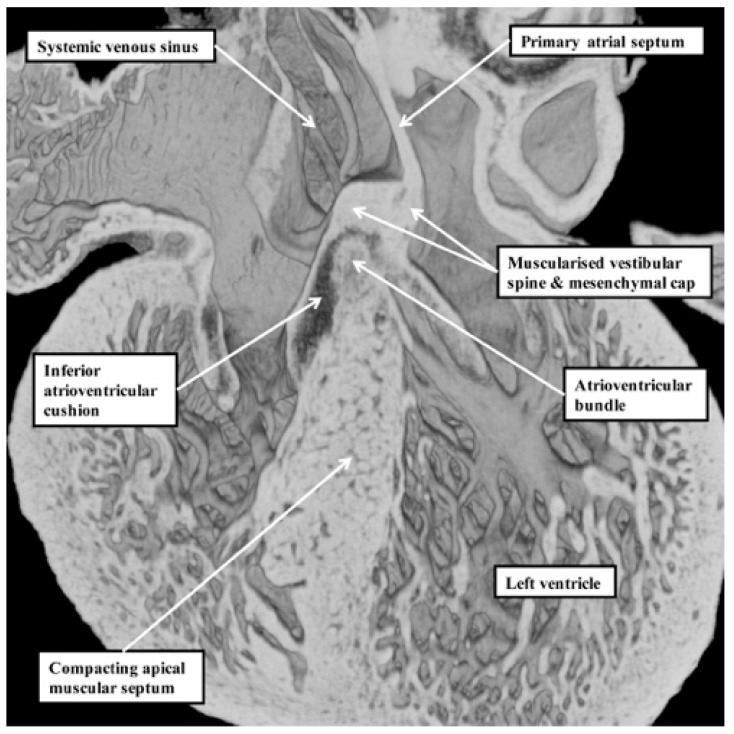
This image is a “four chamber” section through a dataset obtained from a developing mouse embryo at embryonic day 14.5. The location of the atrioventricular bundle, sandwiched between the crest of the muscular ventricular septum and the insulating tissues derived from the atrioventricular cushions, shows that there is no ventricular “septum of the atrioventricular canal” [8]. As can be seen, the structure responsible for dividing the initially common atrioventricular canal—the vestibular spine [9,10]—forms the antero-inferior buttress of the atrial septum.

Our developmental findings are also pertinent to the suggestion that there is within the normal muscular ventricular septum a component derived from “the septum of the atrioventricular canal” [6]. It is now well established that such a septal component is, indeed, key to the separation of the initially common atrioventricular canal into the right and left atrioventricular junctions. This entity is the vestibular spine. Along with the mesenchymal cap carried on the leading edge of the primary atrial septum, the spine is muscularised to form the antero-inferior buttress of the atrial septum [8]. The spine was initially described in the 19th century [9], when it was called the “spina vestibuli”. Its importance was re-discovered by Snarr and colleagues, who described the entity as the dorsal mesenchymal protrusion [10]. Subsequent to the completion of septation, however, the myocardialised entities form part of the atrial, rather than the ventricular, septum. It is the location of the atrioventricular bundle, sandwiched between the crest of the muscular ventricular septum and the insulating tissues of the atrioventricular junctions, which confirms that in the normal heart, there is no ventricular “septum of the atrioventricular canal” (Figure 7) [11].

## 3. Discussion

**Figure 8:**
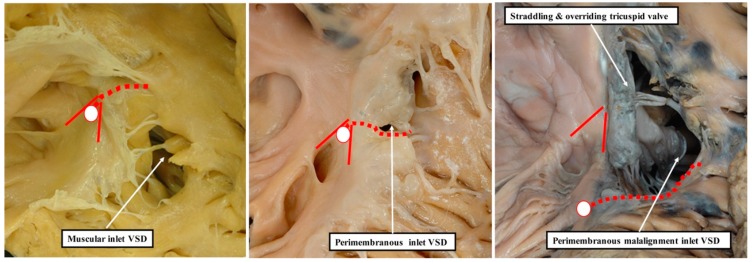
These images show that a description of geography does not provide the significant information regarding the phenotypic variability of ventricular septal defects. All three defects are properly classified as inlet defects. In this regard, we do not underestimate the importance of describing this geographical feature. It is only when the borders are examined, however, that the defect shown in the **left** panel is identified as a muscular defect, and the defects in the **middle** and **right**-hand panels are identified as being perimembranous. Further examination of the perimembranous defects on the basis of their borders then reveals that the heart shown in the **right**-hand panel has malalignment between the atrial septum and the muscular ventricular septum. It is the information derived on the basis of borders, therefore, that provides the crucial clinical information regarding the location of the atrioventricular conduction axis, shown as the white oval with red borders and the dotted red line (see also Reference 15). The red lines without dots show the location of the triangle of Koch.

Poelmann and Gittenberger-de Groot [1] are correct in asserting that the so-called “aortopulmonary septal complex” is better termed the “outflow tract septal complex”. This fact is of particular importance for the understanding of postnatal anatomy and has significance for the categorisation of ventricular septal defects [3]. This is because when development proceeds normally, the entities which divide the developing outflow tract, and which initially have a septal location, subsequently lose this septal function. This occurs concomitantly with the development of the discrete walls of the intrapericardial arterial trunks, the separation of the aortic and pulmonary roots, and the formation of the free-standing muscular subpulmonary infundibulum. As suggested above, these findings have implications far beyond the understanding of normal postnatal anatomy. The fact that the proximal outflow cushions initially muscularise to produce a septum between the components of the proximal outflow tract, but that with normal development the muscularised tissues subsequently become the free-standing muscular subpulmonary infundibulum, is key to arbitrating ongoing discussions regarding the optimal means of categorising ventricular septal defects in the clinical setting [3]. Thus, the findings show that the notion that the normal muscular ventricular septum has a component derived from the “conus” has no developmental foundation. The same goes for the alleged “septum of the atrioventricular canal”. The definitive ventricular septum has only apical muscular and fibrous components, the fibrous part usually being described as the membranous septum. Our initial studies of the anatomy of ventricular septal defects had indicated that all could be categorised, according to the nature of their borders, into those abutting the remnants of the membranous septum, those embedded within the apical muscular septum, or those reflecting the lack of muscularisation of the proximal outflow cushions [12]. The findings as described by Poelmann and Gittenberger-de Groot [1] provide further evidence in support of this concept. They can be interpreted to endorse the notion that it is the borders of ventricular septal defects that serve best to define their phenotypic differences [13]. This is because it is not possible, when using geography as the starting point for categorisation, to show these crucial phenotypic differences. This potential deficiency of beginning categorisation on the basis of geography is well demonstrated by considering the defects that open to the inlet of the right ventricle (Figure 8). 

**Figure 9:**
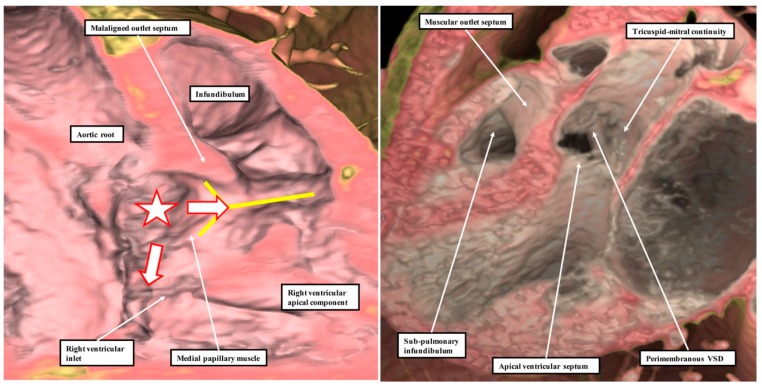
These images are taken from a computerised tomographic dataset made from a patient with a ventricular septal defect (VSD) opening centrally at the base of the right ventricle, as shown by the white star with red borders in the **left**-hand panel. As is then shown by the white arrows with red borders, however, the defect is sufficiently large to extend so as to open in part to the inlet of the right ventricle, but also to the right ventricular outlet. It opens to the outlet component through the limbs of the septal band, shown by the yellow “Y”. It is difficult, therefore, when assessing its geographical categorisation, to determine whether the defect should be deemed to be inlet, central, or outlet. It is better described as being confluent. The view from the left ventricle, as seen in the **right**-hand panel, shows that there is no question but that the defect is perimembranous, since it is bordered postero-inferiorly by fibrous continuity between the leaflets of the tricuspid and mitral valves. Note also that, as seen in the **left**-hand panel, the muscularised proximal cushions can still be recognised as producing a malaligned myocardial outlet septum in this setting of deficient ventricular septation.

It is knowledge of their borders, rather than their geographical location, which provides the crucial clinical information regarding the disposition of the atrioventricular conduction axis [14]. As we show in Figure 9, these borders can now be demonstrated with precision by interrogation during life of computed tomographic datasets. 

**Figure 10:**
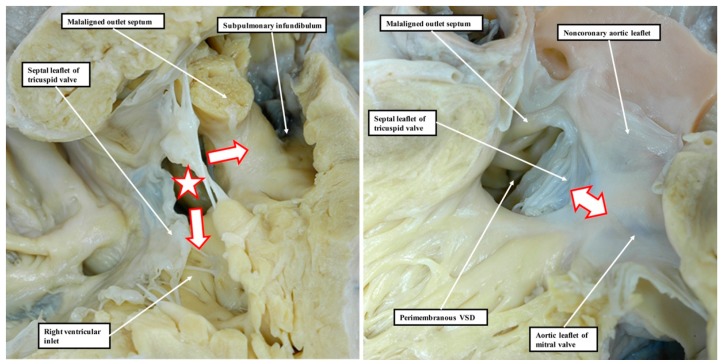
These images show a perimembranous defect that opens centrally at the base of the right ventricle but then, as is also the case for the defect shown in Figure 9, extends so as to open into both the inlet and outlet ventricular components. The **left**-hand panel shows the view from the right side. The defect is marked by the white star with red borders, with the white arrows with red borders showing the extension to the inlet and outlet components of the right ventricle. The **right**-hand panel shows the view from the left side. The defect is shielded by the septal leaflet of the tricuspid valve. An extensive area of fibrous continuity incorporating the atrioventricular component of the membranous septum forms the postero-inferior margin of the defect (double-headed white arrow with red borders). Note that, as in the defect shown in Figure 9, there is malalignment of the muscular outlet septum, which is recognisable as a discrete septal entity. It is deviated into the outlet of the right ventricle in an antero-cephalad fashion.

The defect shown in Figure 9 is then the more important, since it extends so as to open not only to the outlet of the right ventricle in the presence of antero-cephalad malalignment of the muscular outlet septum, but also to the right ventricular inlet. We have previously identified such confluent defects in autopsy archives (Figure 10). 

These confluent defects cannot currently be coded within the definitions submitted by the International Nomenclature Society for inclusion in the 11th iteration of the International Classification of Disease [3]. It is recognised within the review “Striving for Consensus” [3], nonetheless, that lesions such as those shown in Figure 9 and Figure 10 will likely be included in future revisions of the classification. As is stated in the summary to this document, “it is meant to be an organic and continuously evolving classification system that will likely change with field testing and advances in scientific knowledge”. We submit that the images we provide in our review contribute to the anticipated “advances in scientific knowledge”. We also submit that only when data of this kind are taken into account will it prove possible eventually to determine whether geography or borders, both of importance, is the preferred option for the starting point of categorisation.

## References

[1] Poelmann R.E., Gittenberger-de Groot A.C. (2018). Hemodynamics in cardiac development. J. Cardiovasc. Dev. Dis..

[2] Waldo K., Miyagawa-Tomita S., Kumiski D., Kirby M.L. (1998). Cardiac neural crest cells provide new insight into septation of the cardiac outflow tract: Aortic sac to ventricular septal closure. Dev. Biol..

[3] Lopez L., Houyel L., Colan S.D., Anderson R.H., Beland M.J., Aiello V.D., Bailliard F., Cohen M.S., Jacobs J.P., Kurosawa H. (2018). Classification of Ventricular Septal Defects for the Eleventh Iteration of the International Classification of Diseases—Striving for Consensus: A Report From the International Society for Nomenclature of Paediatric and Congenital Heart Disease. Ann. Thorac. Surg..

[4] Mori S., Tretter J.T., Spicer D.E., Anderson R.H. (2019). What is the real cardiac anatomy?. Clin. Anat..

[5] Mohun T.J., Weninger W.J. (2011). Imaging heart development using high-resolution episcopic microscopy. Curr. Opin. Genet. Dev..

[6] Van Praagh R., Geva T., Kreutzer J. (1989). Ventricular septal defects: How shall we describe, name and classify them?. J. Am. Coll. Cardiol..

[7] Merrick A.F., Yacoub M.H., Ho S.Y., Anderson R.H. (2000). Anatomy of the muscular subpulmonary infundibulum with regard to the Ross procedure. Ann. Thorac. Surg..

[8] Anderson R.H., Mohun T.J., Brown N.A. (2015). Clarifying the morphology of the ostium primum defect. J. Anat..

[9] His W. (1885). Anatomie Menschlicher Embryonen.

[10] Snarr B.S., O’Neal J.L., Chintalapudi M.R., Wirrig E.E., Phelps A.L., Kubalak S.W., Wessels A. (2007). Isl1 expression at the venous pole identifies a novel role for the second heart field in cardiac development. Circ. Res..

[11] Lamers W.H., Wessels A., Verbeek F.J., Moorman A.F.M., Virágh S., Wenink A.C.G., Gittenberger-de Groot A.C., Anderson R.H. (1992). New findings concerning ventricular septation in the human heart. Implications for maldevelopment. Circulation.

[12] Soto B., Becker A.E., Moulaert A.J., Lie J.T., Anderson R.H. (1980). Classification of ventricular septal defects. Br. Heart J..

[13] Anderson R.H., Spicer D.E., Mohun T.J., Hikspoors J.P.J.M., Lamers W.H. (2019). Remodelling of the embryonic interventricular communication in regard to the description and classification of ventricular septal defects. Anat. Rec..

[14] Spicer D.E., Anderson R.H., Backer C.L. (2013). Clarifying the surgical morphology of inlet ventricular septal defects. Ann. Thorac. Surg..

